# Supplementary material for: The genome sequence of a Chequered Beetle, Tillus elongatus (Linnaeus, 1758) (Coleoptera: Cleridae)
Source: Wellcome Open Res. 2025 Oct 13;10:575. [Version 1] doi: 10.12688/wellcomeopenres.24678.1 (PMC12640499; doi:10.12688/wellcomeopenres.24678.1)
Supplement: Peer review history

# Reviewer report: Reviewer response for version 1

**DOI:** 10.21956/wellcomeopenres.27193.r136862

**Published:** 2025-11-24

**Authors:** Sivasankaran Kuppusamy

**Affiliations:** 1 Loyola College, Chennai, tamilnadu, India

The authors sequenced the genome of *Tillus elongatus* (Linnaeus, 1758). Authors have assembled the genome sequence and received a total length of 1,719.08 Mb. They received 10 chromosomal pseudomolecules through scaffolding.

**Minor comments on the manuscript**

 In the background of the manuscript, the first sentence *Tillus elongatus *(Linnaeus, 1758) belongs in the Family. The first letter of the “Family” can be a small letter.

 The authors consistently used the whole form of the genus name *Tillus* throughout the text. The genus name can be used in full form first time and the can be a short form like *Tillus* *elongatus.*

 The genome was not annotated in this genome sequencing. Any reason? Because protein-coding genes, non-coding genes and gene transcripts can be observed through the genome annotation.

 The research article was well prepared and the manuscript meets the necessary scientific standard and is suitable for indexing

Are sufficient details of methods and materials provided to allow replication by others?

Yes

Is the rationale for creating the dataset(s) clearly described?

Yes

Are the datasets clearly presented in a useable and accessible format?

Yes

Are the protocols appropriate and is the work technically sound?

Yes

Reviewer Expertise:

Phylogenetic analysis of Noctuoidea moths using mitochondrial genome sequence

I confirm that I have read this submission and believe that I have an appropriate level of expertise to confirm that it is of an acceptable scientific standard.

# Reviewer report: Reviewer response for version 1

**DOI:** 10.21956/wellcomeopenres.27193.r138146

**Published:** 2025-11-22

**Authors:** Krystyna Nadachowska-Brzyska

**Affiliations:** 1 Uppsala University, Uppsala, Sweden; 2 Uniwersytet Jagiellonski w Krakowie Instytut Nauk o Srodowisku, Kraków, Lesser Poland Voivodeship, Poland

This report presents the genome assembly of a male Tillus elongatus individual. This is the first genome for the genus Tillus, and one of only two available for the Clerida family. This makes it a valuable resource not only for this family, but also more generally for the underrepresented (in the number of available genome assemblies) Coleaptera group.

 Following the standards and presentation style of Wellcome Open Research reports, it provides sufficient details on the methodology, assembly statistics, and other results. As far as I can tell, it is a good chromosomal-level assembly. I only have minor comments.

 1. It would be helpful to include a small map or provide a link to an existing resource showing the geographical distribution of T. elongatus. This would make it much easier to understand than reading about the distribution alone.

 2. The description of the species morphology reads like a popular science description. I would appreciate a more formal description.

 3. Is classical cytogenetic karyotyping available for the species?

 4. There is a missing space after 'UK' in the sentence:

 '... collected from Kelling Heath, England, UK (latitude 52.93, longitude 1.12)'.

 5. The authors state that 'the genome will be annotated using available RNA-Seq data and presented through the Ensembl pipeline at the European Bioinformatics Institute'.

 Shouldn't reports include annotations at publication?

Are sufficient details of methods and materials provided to allow replication by others?

Yes

Is the rationale for creating the dataset(s) clearly described?

Yes

Are the datasets clearly presented in a useable and accessible format?

Yes

Are the protocols appropriate and is the work technically sound?

Yes

Reviewer Expertise:

population genetics

I confirm that I have read this submission and believe that I have an appropriate level of expertise to confirm that it is of an acceptable scientific standard.

# Reviewer report: Reviewer response for version 1

**DOI:** 10.21956/wellcomeopenres.27193.r138143

**Published:** 2025-11-11

**Authors:** Geoff S Oxford

**Affiliations:** 1 University of York, Heslington, UK

The background is short but informative. It would be useful if field photographs of adult specimens with a red and a dark (often black) pronotum (e.g. https://www.eakringbirds.com/eakringbirds2/insectinfocustilluselongatus.htm) and also of larvae and pupae (e.g. https://www.shutterstock.com/search/tillus-elongatus). My only other comment is that a space is needed under 'Sample acquisition and DNA barcoding', 2nd line between 'UK' and '(latitude...'. It is not within my expertise to judge the technical aspects, which are standard DToL procedures.

Are sufficient details of methods and materials provided to allow replication by others?

Yes

Is the rationale for creating the dataset(s) clearly described?

Yes

Are the datasets clearly presented in a useable and accessible format?

Yes

Are the protocols appropriate and is the work technically sound?

Yes

Reviewer Expertise:

I have worked on several species of beetle and have a background in population genetics, including molecular aspects. However, I don't have the expertise to judge what are standard DToL procedures.

I confirm that I have read this submission and believe that I have an appropriate level of expertise to confirm that it is of an acceptable scientific standard.
